# Apolipoprotein‐E genotyping in formalin‐fixed and paraffin‐embedded post‐mortem brain tissue

**DOI:** 10.1111/bpa.13243

**Published:** 2024-01-25

**Authors:** James Minshull, Yvonne Davidson, Federico Roncaroli, Andrew C. Robinson

**Affiliations:** ^1^ Faculty of Biology Medicine and Health, School of Biological Sciences, Division of Neuroscience The University of Manchester Manchester UK; ^2^ Geoffrey Jefferson Brain Research Centre Manchester Academic Health Science Centre (MAHSC) Manchester UK; ^3^ Northern Care Alliance Salford Royal Hospital Salford UK

**Keywords:** Alzheimer's disease, *APOE*, genotyping techniques, neurodegenerative diseases, PCR

## Abstract

Formalin‐fixed paraffin‐embedded (FFPE) brain tissue held in tissue banks constitutes a valuable research resource, especially when associated with clinical annotations and longitudinal psychometric testing. Apolipoprotein‐E (APOE) genotyping is important to fully characterise this resource, however older FFPE tissue may not be suitable for genotyping. We performed polymerase chain reaction restriction fragment length polymorphism (PCR‐RFLP) assays on DNA extracted from post‐mortem FFPE brain tissue ranging from 2‐19 years old. A maximum of three years in paraffin was determined for robust APOE genotyping of FFPE tissue using PCR‐RFLP which may suggest prolonged storage of fixed tissue as FFPE blocks may have deleterious effects on DNA.

## Graphical abstract

Formalin‐fixed paraffin‐embedded brain tissue held in tissue banks constitutes a valuable research resource, especially when associated with clinical annotations and longitudinal psychometric testing. Apolipoprotein‐E (APOE) genotyping is important to fully characterize this resource. A maximum of three years in paraffin was determined for robust APOE genotyping of FFPE tissue using PCR‐RFLP.

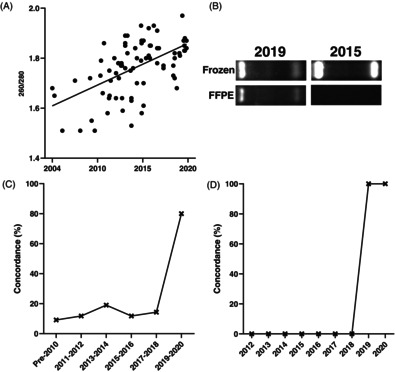

## ETHICS STATEMENT

The study was approved by Manchester Brain Bank Management Committee (REC reference 19/NE/0242). Under conditions agreed with the Research Ethics Committee, The Manchester Brain Bank can supply tissue or data to researchers, without requirement for researchers to apply individually to the REC for approval.

Formalin fixation, dehydration, paraffin wax infusion, and embedding of human tissue is standard practice in histopathology as it allows for long‐term storage of specimens at ambient temperatures and preserves tissue for microscopic examination and immunohistochemistry. However, DNA and RNA are damaged in formalin‐fixed and paraffin‐embedded (FFPE) tissue and may further degrade over time [1].

Tissue banks worldwide store thousands of FFPE tissue blocks. These specimens constitute a valuable research resource, especially when associated with clinical annotations and longitudinal psychometric testing. Such resources are often underutilized due to the misconception that preservation of post‐mortem tissue is suboptimal and FFPE processing limits genetic characterization. For instance, brain banks that are part of the United Kingdom Brain Bank Network predominantly collect from donors with neurodegenerative conditions but only 17% of cases have recorded apolipoprotein‐E (*APOE*) genotype (UK Brain Banks Network: https://brainbanknetwork.ac.uk).

Knowledge of *APOE* genotype is relevant to neuropathological assessment and genotype–phenotype correlations. More thoroughly characterized cases will also be more informative to researchers who wish to access cases stored in brain banks. *APOE* is implicated in Alzheimer's disease (AD) and other neurodegenerative diseases. Two single nucleotide variants (SNVs) of *APOE*—388 T > C and 526C > T determine the three alleles ε2, ε3, and ε4 [2]. Around 60% of European, North American, and Australian populations have the ε3/ε3 genotype, which has a neutral effect on the risk of developing AD [3]. A ε3/ε4 genotype is associated with a four‐fold increased risk of AD, while ε4/ε4 imparts a 12‐fold increased risk [4]. The ε2 allele is regarded as neuroprotective against AD. The presence of one ε2 allele decreases risk of AD by around four times [5]. However, ε2 has been associated with cerebral amyloid angiopathy [6].

To maximize the use of tissue bank holdings, we sought to investigate the feasibility of *APOE* genotyping from FFPE brain samples and to provide guidance on the viability of FFPE specimens that have been stored for many years.

The DNA from 85 archival FFPE samples from the cerebellum (mean PMD 82 ± 36 h, range 25–182.5 h), that had been preserved in wax for up to 19 years at the Manchester Brain Bank (MBB) was extracted with a widely used extraction kit (Qiagen's QIAamp DNA FFPE Tissue Kit, Hilden, Germany, QIA) with polymerase chain reaction (PCR) and restriction fragment length polymorphism (RFLP) whereby samples underwent endonuclease restriction and genotyping using gel electrophoresis. Matched frozen tissue and blood also underwent concurrent DNA extraction and PCR using the REDExtract‐N‐Amp tissue PCR kit (Merck, Darmstadt, Germany, XNAT) and REDExtract‐N‐Amp blood PCR kit (XNAB). All cerebellar FFPE extractions were carried out in December 2022 and frontal FFPE extractions in November 2023, and relevant statistical analyses were carried out in relation to these dates. The diagnoses of the analyzed samples are summarized in Table 1.

**TABLE 1:** Pathological characteristics of the analyzed cohort.

Pathology	*N*
Frontotemporal lobar degeneration	26
TDP‐43 pathology	20
Tau pathology	6
Alzheimer's disease	20
Progressive supranuclear palsy	9
Corticobasal degeneration	7
Motor neurone disease	7
Dementia with lewy bodies	6
Multiples system atrophy	3
Ageing related changes	3
Parkinson's disease	2
Small vessel disease	2
Total	85

The methodology was optimized using 1:10 dilution of extracted DNA diluted in distilled water before loading into PCR (approximately 50 ng DNA). At this concentration, the interpretation of *APOE* genotypes using PCR‐RFLP improved. DNA concentrations and 260/280 ratios for the whole cohort are available in the supplementary materials.

**FIGURE 1:**
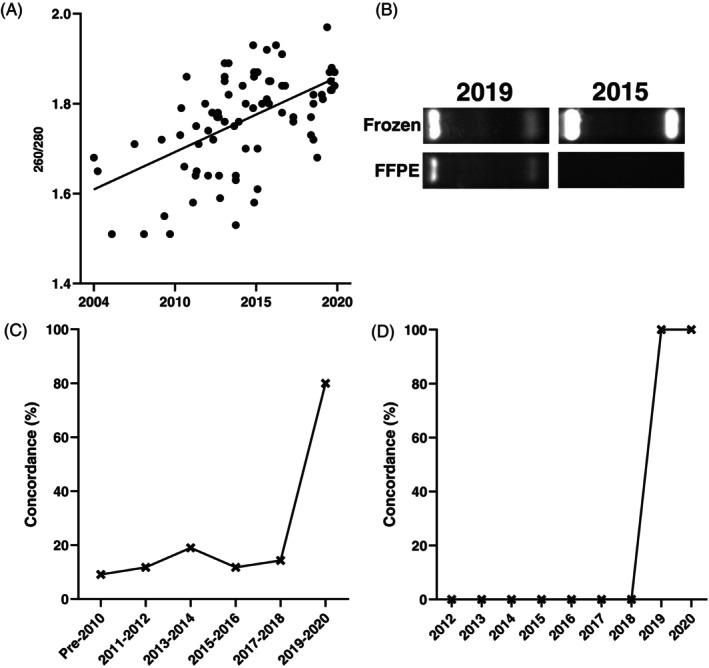
(A) Correlation between time since FFPE processing with QIA and DNA quality as measured by 260/280 ratio, *R* ^2^ = 0.30. (B) Example gel images of matched DNA samples derived from both frozen and FFPE tissue from years 2019 and 2015. 2/2 genotype shown. (C) Percentage concordance of QIA‐derived *APOE* genotype in cerebellar tissue when compared with frozen‐derived genotype over time. (D) Percentage concordance of QIA‐derived *APOE* genotype in frontal tissue when compared with frozen‐derived genotype over time.

Positive yet weak associations were found between sample quality (260/280) and time since routine FFPE processing was performed (Figure 1A, *R* ^2^ = 0.3), suggesting that an older FFPE sample will, on average, produce lower‐quality DNA. However, DNA 260/280 ratios were highly variable. No association was found between DNA quality and post‐mortem delay (Supplementary Figure 1).

Visualization of *APOE* genotypes with RFLP gel electrophoresis showed that older FFPE specimens produced multiple non‐specific bands or no bands at all (Figure 1B). For the purposes of our analyses, any results that were ambiguous were counted as negative. When compared with the date of routine processing, concordance with genotyping from frozen tissue was 100% for samples processed in 2020 (*n* = 2) and 80% for samples processed between 2019 and 2020 (*n* = 10). Samples processed prior to 2019 showed *APOE* genotyping accuracy of approximately 13% (*n* = 75) (Figure 1C). No accurate *APOE* genotype was obtained from any FFPE samples processed prior to October 2010. Concordance between frozen tissue‐derived DNA and blood‐derived DNA was 97.6%. The most common accurately identified *APOE* genotype in FFPE tissue was ε3/ε3 (Supplementary Table 2).

Cerebellar cortex was used in this study to optimize the protocol because of its high neuronal density, which allowed us to explore the feasibility of *APOE* sequencing from FFPE specimens. We then validated the protocol on DNA from the superior frontal gyrus. A similar non‐linear decrease in concordance between FFPE and frozen tissue was observed at approximately 4 years after processing (Figure 1D), whilst 260/280 ratios showed a more linear negative association with time since processing (Supplementary Figure 2).

We observed that FFPE‐derived DNA from older blocks can show high 260/280 ratios but its integrity is insufficient to provide accurate *APOE* genotyping. Our results demonstrate that *APOE* genotype can be accurately determined from archival FFPE samples using RFLP when the tissue has been in paraffin for less than 4 years. Length of formalin fixation prior to paraffin embedding had a marginal negative impact on DNA quality and genotyping suggesting that brains stored in formalin can still be suitable for APOE genotyping (Supplementary Figure 3).

The decline in accuracy of *APOE* genotyping after 4 years in paraffin was considerable. 250 bp has been suggested as the limit of amplimer length for accurate FFPE DNA extraction and amplification [7]. As the longest fragment produced by our PCR‐RFLP method was 104 bp, clear *APOE* genotypes should have been detectable using this method. Poor PCR results in older samples may have been caused by inhibition of the DNA polymerase enzyme by small DNA fragments produced by excess formalin [8].

FFPE processing may introduce artefactual changes to DNA such as deamination of cytosine to uracil [9]. As both *APOE* primers are guanine‐rich (Supplementary Table 1) and the HhaI restriction enzyme recognizes GCGC sites, this may have caused reduced PCR‐RFLP efficacy in older samples as DNA binding sites were mutated. The lack of interpretable genotypes in older samples could be a result of deleterious effects of FFPE processing and storage, representing a potential limitation of the use of FFPE tissue.

Although genotyping with RFLP is a lesser‐used method in current genetic research, it remains a reliable and cheap technique that is useful for large‐scale application in tissue banks. More advanced techniques such as qPCR with TaqMan probe which can detect *APOE* allele‐specific SNVs [10] may be able to provide more robust genotyping of older FFPE‐derived DNA but can be more expensive. Further optimization of PCR‐RFLP through alterations to PCR timings and concentrations of reagents such as DNA polymerase and deoxynucleotide triphosphates could be pursued to improve the performance of FFPE‐derived DNA in PCR‐RFLP [8].

All samples in this study were acquired from the tissue archives at MBB and, therefore, were subject to MBB standard processing protocols. However, changes in suppliers of laboratory consumables over time, such as paraffin and solvents, may have impacted on FFPE tissue processing and could not be controlled for in this study. Furthermore, residual formalin and the addition of additives to paraffin, such as plastic polymers and solvents, may have interfered with DNA extraction. Specifically, DNA quality and our ability to obtain meaningful *APOE* genotypes could have been affected by technical variables that are specific to MBB. Further work using FFPE tissue from across several tissue banks may be required to assess any site‐specific variables.

Despite these limitations, our study provides insights into the use of FFPE samples for *APOE* genotyping of brain samples that do not have associated frozen tissue or blood samples. We recommend that *APOE* allele status is investigated soon after paraffin embedding or within 3 years as these specimens are likely to provide high‐quality DNA. Analysis can also be attempted after long‐term storage, but results are variable. PCR‐RFLP assays remain a reliable and affordable technique for retrospective assessment of *APOE* genotype.

## AUTHOR CONTRIBUTIONS

JM performed sample preparation, performed statistical analyses, and wrote the manuscript. YD performed PCR‐RFLP analyses and assisted in preparation of the manuscript. FR designed the study, finalized neuropathological diagnoses, and assisted in preparation of the manuscript. AR devised and designed the study and assisted in preparation of the manuscript.

## CONFLICT OF INTEREST STATEMENT

The authors confirm that they have no conflicts of interest to disclose.

## Supporting information


**Supplementary Figure 1:** Post‐mortem delay (hours) versus 260/280 ratio (cerebellar), *R* ^2^ = 0.00001, simple linear regression.
**Supplementary Figure 2:** Days since processing versus 260/280 ratios for FFPE‐derived DNA from superior frontal gyrus, *R* ^2^ = 0.30, simple linear regression.
**Supplementary Figure 3:** (A) Formalin fixation (days) versus DNA quality (260/280), *R* ^2^ = 0.31, simple linear regression. (B) Cerebellar FFPE‐derived genotype and frozen/blood‐derived genotype concordance versus formalin fixation time (days), unpaired *t*‐test, ns.
**Supplementary Table 1:** *APOE* forward and reverse primers for PCR.
**Supplementary Table 2:** *N* of each interpretable genotype detected for the full cohort using QIA.
**Supplementary Table 3:** Concordance between cerebellar FFPE‐derived genotype and frozen tissue‐derived genotype per pathology.
**Supplementary Table 4:** Concentration and 260/280 ratios for the full cohort.

## Data Availability

The datasets used and/or analysed during the current study are available from the corresponding author on reasonable request.
