# Supplementary material for: Apolipoprotein‐E genotyping in formalin‐fixed and paraffin‐embedded post‐mortem brain tissue
Source: Brain Pathol. 2024 Jan 25;34(5):e13243. doi: 10.1111/bpa.13243 (PMC11328341; doi:10.1111/bpa.13243)
Supplement: Supplementary file 1 — Supplementary Figure 1: Post‐mortem delay (hours) versus 260/280 ratio (cerebellar), R 2 = 0.00001, simple linear regression. Supplementary Figure 2: Days since processing versus 260/280 ratios for FFPE‐derived DNA from superior frontal gyrus, R 2 = 0.30, simple linear regression. Supplementary Figure 3: (A) Formalin fixation (days) versus DNA quality (260/280), R 2 = 0.31, simple linear regression. (B) Cerebellar FFPE‐derived genotype and frozen/blood‐derived genotype concordance versus formalin fixation time (days), unpaired t‐test, ns. Supplementary Table 1: APOE forward and reverse primers for PCR. Supplementary Table 2: N of each interpretable genotype detected for the full cohort using QIA. Supplementary Table 3: Concordance between cerebellar FFPE‐derived genotype and frozen tissue‐derived genotype per pathology. Supplementary Table 4: Concentration and 260/280 ratios for the full cohort. [file BPA-34-e13243-s001.docx]

Supplementary Figure 1: Post-mortem delay (hours) vs 260/280 ratio (cerebellar), R^2^ = 0.00001, simple linear regression.

Supplementary Figure 2: Days since processing vs 260/280 ratios for FFPE-derived DNA from superior frontal gyrus, R^2^=0.30, simple linear regression.

Supplementary Figure 3: A) Formalin fixation (days) vs DNA quality (260/280), R^2^ = 0.31, simple linear regression. B) Cerebellar FFPE-derived genotype and frozen/blood-derived genotype concordance vs formalin fixation time (days), unpaired t-test, ns.

**B**

**A**

| ApoE rev | 5’ - GGC GCT CGC GGA TGG CGC TGA G - 3’ |
| --- | --- |
| ApoE for | 5’ - GCA CGG CTG TCC AAG GAG CTG GAG GC - 3’ |

Supplementary Table 1: *APOE* forward and reverse primers for PCR.

| Genotype | N |
| --- | --- |
| 22 | 0 |
| 23 | 3 |
| 24 | 0 |
| 33 | 10 |
| 34 | 3 |
| 44 | 4 |

Supplementary Table 2: N of each interpretable genotype detected for the full cohort using QIA.

| **Pathology** | **N** | **No. Concordances** | **% Concordance** |
| --- | --- | --- | --- |
| Frontotemporal Lobar Degeneration | 26 | 3 | 11.5 |
| TDP-43 Pathology | 20 | 3 | 15.0 |
| Tau Pathology | 6 | 0 | 0.0 |
| Alzheimer's Disease | 20 | 5 | 25.0 |
| Progressive Supranuclear Palsy | 9 | 0 | 0.0 |
| Corticobasal Degeneration | 7 | 4 | 57.1 |
| Motor Neurone Disease | 7 | 1 | 14.3 |
| Dementia with Lewy Bodies | 6 | 1 | 16.7 |
| Multiples System Atrophy | 3 | 1 | 33.3 |
| Ageing Related Changes | 3 | 1 | 33.3 |
| Parkinson's Disease | 2 | 2 | 100.0 |
| Small Vessel Disease | 2 | 0 | 0.0 |

Supplementary Table 3: Concordance between cerebellar FFPE-derived genotype and frozen tissue-derived genotype per pathology.

|  | Cerebellum | | Superior Frontal Gyrus | |
| --- | --- | --- | --- | --- |
| Case No. | Concentration (ng/µl) | 260/280 | Concentration (ng/µl) | 260/280 |
| 1 | 23 | 1.68 |  |  |
| 2 | 30 | 1.65 |  |  |
| 3 | 33 | 1.51 |  |  |
| 4 | 78 | 1.71 |  |  |
| 5 | 26 | 1.51 |  |  |
| 6 | 140 | 1.72 |  |  |
| 7 | 94 | 1.55 |  |  |
| 8 | 44 | 1.51 |  |  |
| 9 | 87 | 1.73 |  |  |
| 10 | 132 | 1.79 |  |  |
| 11 | 102 | 1.66 |  |  |
| 12 | 184 | 1.86 |  |  |
| 13 | 48 | 1.58 |  |  |
| 14 | 46 | 1.64 |  |  |
| 15 | 99 | 1.75 |  |  |
| 16 | 30 | 1.65 |  |  |
| 17 | 130 | 1.71 |  |  |
| 18 | 274 | 1.8 |  |  |
| 19 | 98 | 1.59 |  |  |
| 20 | 217 | 1.64 |  |  |
| 21 | 160 | 1.74 | 197 | 1.41 |
| 22 | 129 | 1.78 |  |  |
| 23 | 81 | 1.78 |  |  |
| 24 | 102 | 1.72 |  |  |
| 25 | 147 | 1.77 |  |  |
| 26 | 169 | 1.77 |  |  |
| 27 | 52 | 1.78 |  |  |
| 28 | 92 | 1.77 |  |  |
| 29 | 58 | 1.64 | 109 | 1.51 |
| 30 | 107 | 1.76 |  |  |
| 31 | 73 | 1.86 |  |  |
| 32 | 92 | 1.85 |  |  |
| 33 | 73 | 1.89 |  |  |
| 34 | 263 | 1.82 | 154 | 1.47 |
| 35 | 95 | 1.89 |  |  |
| 36 | 104 | 1.53 |  |  |
| 37 | 203 | 1.75 |  |  |
| 38 | 71 | 1.63 |  |  |
| 39 | 58 | 1.64 |  |  |
| 40 | 205 | 1.76 | 82 | 1.76 |
| 41 | 65 | 1.84 |  |  |
| 42 | 266 | 1.84 | 191 | 1.54 |
| 43 | 133 | 1.8 |  |  |
| 44 | 173 | 1.7 |  |  |
| 45 | 173 | 1.79 |  |  |
| 46 | 47 | 1.93 |  |  |
| 47 | 131 | 1.86 |  |  |
| 48 | 77 | 1.87 |  |  |
| 49 | 125 | 1.58 |  |  |
| 50 | 180 | 1.86 | 26 | 1.64 |
| 51 | 123 | 1.87 |  |  |
| 52 | 83 | 1.61 |  |  |
| 53 | 66 | 1.7 | 65 | 1.70 |
| 54 | 75 | 1.8 |  |  |
| 55 | 63 | 1.92 |  |  |
| 56 | 116 | 1.81 |  |  |
| 57 | 278 | 1.8 |  |  |
| 58 | 131 | 1.85 |  |  |
| 59 | 394 | 1.85 | 41 | 1.61 |
| 60 | 59 | 1.93 | 100 | 1.56 |
| 61 | 120 | 1.88 |  |  |
| 62 | 44 | 1.84 |  |  |
| 63 | 186 | 1.78 |  |  |
| 64 | 90 | 1.91 |  |  |
| 65 | 159 | 1.84 |  |  |
| 66 | 124 | 1.68 |  |  |
| 67 | 130 | 1.77 | 78 | 1.76 |
| 68 | 122 | 1.77 | 99 | 1.71 |
| 69 | 88 | 1.76 | 169 | 1.47 |
| 70 | 106 | 1.73 | 113 | 1.43 |
| 71 | 50 | 1.77 |  |  |
| 72 | 198 | 1.72 |  |  |
| 73 | 225 | 1.8 |  |  |
| 74 | 51 | 1.82 | 66 | 1.55 |
| 75 | 63 | 1.68 |  |  |
| 76 | 244 | 1.82 |  |  |
| 77 | 268 | 1.81 | 121 | 1.59 |
| 78 | 70 | 1.97 |  |  |
| 79 | 173 | 1.87 |  |  |
| 80 | 219 | 1.83 |  |  |
| 81 | 271 | 1.85 |  |  |
| 82 | 154 | 1.88 |  |  |
| 83 | 240 | 1.83 | 83 | 1.89 |
| 84 | 152 | 1.87 | 68 | 1.89 |
| 85 | 224 | 1.84 | 51 | 1.92 |

Supplementary Table 4: Concentration and 260/280 ratios for the full cohort.
